# Effects of Bisphenols on RACK1 Expression and Their Immunological Implications in THP-1 Cells

**DOI:** 10.3389/fphar.2021.743991

**Published:** 2021-09-21

**Authors:** Erica Buoso, Maša Kenda, Mirco Masi, Pasquale Linciano, Valentina Galbiati, Marco Racchi, Marija Sollner Dolenc, Emanuela Corsini

**Affiliations:** ^1^ Università Degli Studi di Pavia, Dipartimento di Scienze del Farmaco, Pavia, Italy; ^2^ University of Ljubljana, Faculty of Pharmacy, Ljubljana, Slovenia; ^3^ Scuola Universitaria Superiore IUSS, Pavia, Italy; ^4^ Università Degli Studi di Milano, Laboratory of Toxicology, Dipartimento di Scienze Politiche ed Ambientali, Milan, Italy

**Keywords:** bisphenols, hormone effects, endocrine-disrupting chemicals (EDCs), RACK1, immune function, immunotoxicity, GPER, AR

## Abstract

Receptor for activated C kinase 1 (RACK1) has an important role in immune activation, and is regulated through a balance between glucocorticoid and androgen levels. We have previously demonstrated that RACK1 expression can serve as a marker for evaluation of immunotoxic profiles of hormone-active substances, such as endocrine-disrupting chemicals (EDCs). In this study, we investigated the effects of three bisphenols (BPA, BPAF, BPS) on RACK1 expression and on the innate immune responses in the THP-1 human promyelocytic cell line, a validated model for this investigation. BPA and BPAF reduced RACK1 promoter transcriptional activity, mRNA expression, and protein levels. However, BPS had the opposite effect. As expected, these results on RACK1 were paralleled by lipopolysaccharide (LPS)-induced interleukin-8 (IL-8) and tumor necrosis factor-α (TNFα) production. Since BPA and BPAF induced RACK1 expression in the presence of glucocorticoid receptor (GR) antagonist mifepristone, a role of G-protein-coupled estrogen receptor (GPER) has been considered due to their known estrogenic profile. Therefore, additional molecular effects of BPA and BPAF were unmasked after treatment with different inhibitors of well-known pivotal players of GPER-mediated signaling. BPA exerted its effects on RACK1 *via* NF-κB, as shown using the NF-κB inhibitor BAY11-7085 and NF-κB-specific luciferase reporter assay. Conversely, BPAF induced RACK1 up-regulation *via* androgen receptor (AR) activation, as confirmed by treatment with AR antagonist flutamide. Indeed, a biased agonism profile for BPA and BPAF for GPER was suggested based on their different binding modes revealed by our molecular docking. Altogether, our data suggest that RACK1 could represent an important target of EDCs and serves as a screening tool for their immunotoxic potential. Furthermore, RACK1 can be exploited to unmask multiple molecular interactions of hormone-active substances to better dissect out their mechanisms of action.

## Introduction

According to the World Health Organization (WHO), an endocrine disrupting chemical (EDC) is “an exogenous substance or mixture that alters function(s) of the endocrine system and consequently causes adverse effects in an intact organism, or its progeny, or (sub)populations” (WHO, 2013). Due to the immunotoxicity concerns of these chemicals, authorities such as the European Food Safety Authority (EFSA) have promoted research and critical interpretation of the effects of EDCs on the immune system (EFSA, 2016). The most extensively researched EDCs are the bisphenols. The bisphenols are used in the production of epoxy resins and polycarbonate plastics, and are thereby incorporated into a wide range of consumer products, such as thermal paper, electronic equipment, toys, water pipes, sports equipment, medical devices, kitchenware, food contact containers, and dental sealants (Ribeiro et al., 2017).

Bisphenol A (BPA) is the most widely used and studied of the bisphenols, and its effects on the immune system at the cellular level were recently reviewed by Nowak et al. (2019). Both *in vitro* and *in vivo* studies have shown the immunotoxic potential of BPA (Nowak et al., 2019). In contrast, some studies have concluded that BPA is likely not toxic to the immune system, including, in particular the National Toxicology Program-conducted Consortium Linking Academic and Regulatory Insights on Toxicity of BPA (CLARITY-BPA) study (Li et al., 2018). However, BPA remains recognized as toxic mostly due to its endocrine activity, and it is therefore being replaced by other bisphenols, such as bisphenols B, C, F, AF, and S. Paradoxically, data on the potential toxicity of these bisphenol substitutes are scarce, with indications that some of them might be even more hazardous than BPA (Pelch et al., 2019). Indeed, Malaisé et al. (2020) showed that both BPA and its analogs have immunotoxic effects *in vivo*. Therefore, there is a growing need to determine the mechanism(s) through which the bisphenols can affect the immune system, and thence to search for molecular biomarkers to develop a method to rapidly predict immunotoxicity of novel bisphenols before they come into widespread use.

RACK1 is a scaffolding protein involved in a variety of signaling pathways, controlling essential cellular processes and important biological events, including cancer and immune response (Buoso et al., 2020a; Corsini et al., 2021). Our published data show that RACK1 expression is tightly correlated with the production of pro-inflammatory cytokines IL-8 and TNF-α, which we previously demonstrated to be dependent on RACK1/PKCβ activation (Corsini et al., 2021). In addition, we provided evidence for the existence of a complex hormonal balance, between glucocorticoids and androgens, in the control of RACK1 expression and immune cells activation, suggesting that RACK1 can be targeted by EDC (Buoso et al., 2017a; Buoso et al., 2017b; Racchi et al., 2017; Buoso et al., 2020b; Buoso et al., 2020c). We demonstrated that glucocorticoid hormones decrease RACK1 expression while androgens and estrogens increase it (Buoso et al., 2017a; Buoso et al., 2017b; Racchi et al., 2017; Buoso et al., 2020b; Buoso et al., 2020c). In this regard, some EDCs have been shown to have effects on RACK1, including the antiandrogens *p,p’-*dichlorodiphenyltrichloroethane (*p,p’-*DDT) and *p,p’*-dichlorodiphenyldichloroethylene (*p,p’*-DDE) (Buoso et al., 2017a), and the estrogen-active compounds diethylstilbestrol (DES) and zearalenone (ZEA) (Buoso et al., 2020b). In the present study, we investigated BPA, BPAF and BPS effects on the regulation of RACK1 expression and their effects on the immune response. Moreover, we also dissected BPA, BPAF and BPS molecular mechanism in order to elucidate their complex biological activity.

## Materials and Methods

### *In silico* Predictions of Bisphenols Binding to Nuclear Receptors

Predictions for the binding of bisphenols to nuclear receptors AR and GR were investigated using two *in silico* tools: Endocrine Disruptome (Kolšek et al., 2014) and VirtualToxLab (Vedani et al., 2012; Vedani et al., 2009). Both of these tools are recommended for identification of EDCs by the EFSA and the European Chemicals Agency (ECA) (Andersson et al., 2018). Endocrine Disruptome uses molecular docking to predict the binding free energies of compounds to nuclear receptors, and it is available at http://endocrinedisruptome.ki.si/ (Kolšek et al., 2014). It uses Docking Interface for Target Systems (DoTS) for docking simulations and AutoDock Vina for docking calculations. Endocrine Disruptome differentiates between the agonist and antagonist conformations of AR and GR. The structures of each of these receptors were first validated using three databases (i.e., active compounds, agonists, antagonists), as can be downloaded as a pdbqt file (Kolšek et al., 2014). The results are returned as predicted binding free energy based on the sensitivity of each receptor structure: low probability (sensitivity >0.75), moderately low probability (0.5 < sensitivity <0.75), moderately high probability (0.25 < sensitivity <0.5) and high probability of binding (sensitivity <0.25) (Kolšek et al., 2014).

VirtualToxLab (version 5.8; Biographics Laboratory 3R, Basel, Switzerland) uses automated flexible molecular docking with Yeti/AutoDock to consider all compound orientations and conformations in the receptor binding site. This is combined with the multidimensional Quantitative Structure-Activity Relationships (mQSAR) software (Quasar) to define compound orientation, position, different solvation, protonation and conformation, for induced-fit models and the tautomeric state. This tool does not consider agonist or antagonist modes of action on AR and GR. The results are given as the predicted toxic potential (0–1, where 1 is most toxic) and the concentration at which the compound is expected to bind to the nuclear receptor (up to 100 µM considered) (Vedani et al., 2009; Vedani et al., 2012).

### Chemicals

BPA (PubChem CID: 6623), BPAF (PubChem CID: 73864), BPS (PubChem CID: 6626), Mifepristone (RU486) (PubChem CID: 55245), Flutamide (PubChem CID: 3397) and esiRNA MISSION^®^ (EHU068001) for GRα were purchased from Sigma Aldrich (St Louis, MO, United States). Lipopolysaccharide (LPS) from *Escherichia coli* serotype 0127:B8 and the cell culture media and supplements were from Sigma Aldrich (St Louis, MO, United States). The mouse anti-human RACK1 monoclonal antibody (sc-17754) was from Santa Cruz Biotechnology (Dallas, TX, United States). The mouse monoclonal anti-β-tubulin antibody (T0198) was from Sigma Aldrich (St Louis, MO, United States). Electrophoresis reagents were from Bio-Rad (Richmond, CA, United States). Chemicals were diluted in dimethyl sulfoxide (DMSO) for 50 mM stock solutions, and sequentially properly diluted.

### Cell Culture

The THP-1 cell line was originally derived from an acute monocytic leukemia patient, and was from American Type Culture Collection (ATCC TIB-202; Manassas, VA, United States). For experiments, THP-1 cells, were diluted to 10^6^ cells/mL in RPMI 1640 without phenol red containing 2 mM L-glutamine, 0.1 mg/ml streptomycin, 100 IU/ml penicillin, gentamycin 10 μg/ml, 50 µM 2-mercaptoethanol, supplemented with 5% dextran-coated charcoal-treated fetal bovine serum (DCC-FBS) and cultured at 37°C in 5% CO_2_ incubator. Preliminary experiments were conducted to identify non-cytotoxic concentrations [cell viability >80% (CV80)]. Cytotoxicity was assessed by propidium iodine staining and the CV80 determined for all compounds. For the different experiments, cells were then treated with increasing concentrations of BPA, BPAF and BPS (0.001–10 μM) or DMSO as vehicle control (final concentration of DMSO in culture medium <0.1%) as detailed in the legends.

### Plasmid DNA Preparation, Transient Transfections, and Luciferase Assays

Plasmids preparation, transient transfections and luciferase assays were performed as previously described (Buoso et al., 2019; Buoso et al., 2020b). The Δ1 reporter plasmid construct has been previously described (Del Vecchio et al., 2009). It was the longest construct available, 2105 nt long, which contains the *RACK1* gene promoter region between nucleotides −1744 and +361, and includes the *glucocorticoid* responsive element (GRE) sequence. The pGL4.32 vector (E8491; Promega, Madison, WI, United States) luciferase-reporter construct plasmid DNA was also used (Fagiani et al., 2020). Plasmids for transfections were purified with the HiSpeed^®^ Plasmid Midi Kit (Qiagen, Valencia, CA, United States). DNA was quantified and assayed for purity using Quantus™ fluorometer (Promega, Madison, WI, United States). Transient transfections were performed in 24-well plates; for each well, 5 × 10^5^ cells were seeded in RPMI 1640 medium without phenol red, and with 5% DCC-FBS, 1% antibiotics and supplemented with 1% L-glutamine. Transfections were carried out using Lipofectamine® 2000 (Invitrogen Carlsbad, CA, United States), following the manufacturer instructions. Each luciferase reporter construct plasmid DNA was co-transfected with the pRL-TK renilla luciferase expressing vector to measure the transfection efficiency (Promega, Madison, WI, United States). During transfection, THP-1 cells were incubated at 37°C in 5% CO_2_, and then treated with the selected compounds for the times and at concentrations specified in figure legends. Cells were then lysed (Passive Lysis Buffer, provided by the Dual-Luciferase Reporter Assay System; Promega, Madison, WI, United States), following the manufacturer specifications. Luminescence was measured with a 20/20n Luminometer (Turner Bio-Systems, Sunnyvale, CA, United States), with 10 s integration time.

### Reverse Transcription Quantitative PCR

Quantitative PCR (qPCR) was performed as previously described (Buoso et al., 2020b). Total RNA was extracted using RNeasy Plus Mini Kit (Qiagen, Valencia, CA, United States), following the manufacturer instructions. For the synthesis of cDNA, 2 µg total RNA was reverse transcribed using high-capacity cDNA archive kits (Applied Biosystems, Foster City, CA, United States), following the manufacturer instructions. *RACK1* gene expression was determined by qPCR using the Taq-Man™ PCR technology. Primers were from Applied Biosystems. PCR reactions were performed in duplicate, according to the standard protocols suggested by the manufacturer. For each PCR reaction, 10 ng total RNA was used. 18S ribosomal RNA was used as an endogenous reference. Quantification of the transcripts was performed according to the 2^−ΔΔCT^ method.

### Immunoblot Analysis

Immunoblot analysis was performed as previously described (Buoso et al., 2020b). *RACK1* expression was determined at the protein level in cell lysates by immunoblot analysis. After the treatments, the cells were harvested, washed with phosphate-buffered saline (PBS) 1X, centrifuged, lysed in 100 µl homogenization buffer (50 mM Tris-HCl pH 7.5, 150 mM NaCl, 5 mM EDTA, 0.5% Triton X-100, protease inhibitor mix) and sonicated for 10 s. Protein content was measured using Bradford assay. Samples for Western blotting were prepared by mixing cell lysates with sample buffer (125 mM Tris-HCl pH 6, 8.4% sodium dodecyl sulfate (SDS), 20% glycerol, 6% β-mercaptoethanol, 0.1% bromophenol) and denatured at 95°C for 5 min. Equivalent amounts of extracted protein (10 μg) were electrophoresed into 10% SDS-PAGE under reducing conditions. The proteins were then transferred to polyvinylidene fluoride (PVDF) membranes (Amersham, Little Chalfont, United Kingdom), blocked in 5% (w/v) bovine serum albumin (BSA), 1X TBS, 0.1% Tween-20 for 1 h with gentle shaking. Proteins were visualized using primary antibodies diluted in 5% (w/v) BSA, 1X TBS, 0.1% Tween-20 for RACK1 (1:1000) and β-tubulin (1:1,000). In all of the experiments, the immunoreactivity was detected using host-specific secondary IgG peroxidase-conjugated antibodies (1:5,000), and developed using enhanced chemiluminescence (Clarity Western ECL blotting substrates, Bio-Rad). Immunoblot images were acquired with Molecular Imager Gel Doc XR (BioRad), and the optical density of the bands was determined using the ImageJ software (W. Rasband, Research Service Branch, National Institute of Mental Health, National Institutes of Health, Bethesda, MD, United States; and Laboratory for Optical and Computational Instrumentation, University of Wisconsin, WS, United States). The relative densities of the bands are expressed as arbitrary units, and are normalized to the control samples run under the same conditions.

### Cytokine Production

Cytokine production was assessed using TNF-α (Invitrogen, Carlsbad, CA, United States) and IL-8 specific sandwich ELISAs (ImmunoTools, Friesoythe, Germany). To induce cytokine secretion, 10 ng/ml LPS was added with the 24 h bisphenol exposure, which was prolonged for 3 h for TNF-α and for 24 h for IL-8 assays. Cell-free supernatants obtained by centrifugation at 300 × g for 5 min were stored at − 80°C until measurement. Limits of detection were IL-8 2.6 pg/ml for IL 8, and 22 pg/ml for TNF-α. Results are expressed in pg/mL (Buoso et al., 2020b).

### Molecular Docking to G-Protein-Coupled Estrogen Receptor

The three-dimensional (3D) structure of GPER, generated by homology modeling, was recovered from the web server GPCR-ITASSER (Zhang and Zhang, 2010; Zhang et al., 2015). The model_1 of the homology model HG ID: HG0714 (C score = −0.64; Estimated TM-score = 0.63 ± 0.13; Estimated RMSD = 8.1 ± 4.4Å) was employed for docking calculation. The three-dimensional structure was prepared for the subsequent structure-based calculations using the Protein Preparation Wizard utility of the Schrödinger suite (Sastry et al., 2013; Schrödinger LLC New York (USA), 2014d). Missing amino acid side chains were rebuilt, and potential atom types and bond connectivity issues into the homology model structure were fixed. Moreover, ionization and tautomerization states potentially present at physiological pH were also calculated with the Epik sub-routine (Shelley et al., 2007; Schrödinger LLC New York (USA), 2014d). Afterward, the pretreated structure was minimized according to the OPLS3e force field. The GPER receptor grid was generated at the center of the putative binding site (Rosano et al., 2016). The chemical structures of BPA and BPAF were designed in ChemDraw, imported into the Maestro software (Schrödinger, LLC, New York, NY (USA), 2014b), and prepared using the LigPrep utility available within the Schrodinger suite (Schrödinger, LLC, New York, NY (USA), 2014c). All of the potential states of ionization and tautomerism at a physiological pH of 7.4 ±0.2 were generated. The prepared bisphenols were subjected to 2,000 minimization steps with MacroModel, using the OPLS3e force field. The ligands thus prepared were finally docked to the protein. The Glide software was used for the docking calculation, using the default settings of the Standard Precision (SP) protocol (Schrödinger, LLC, New York, NY (USA), 2014a). The resulting ligand-protein complexes were ranked by docking score and visually inspected.

### Statistical Analysis

Data are expressed as means ± standard error of mean (SEM) of at least three independent experiments. Statistical analysis was performed using the InStat software, version 7.0 (GraphPad Software, La Jolla, CA, United States). Significant differences were determined using Student’s *t*-tests or analysis of variance (ANOVA), followed, when significant, by an appropriate *post hoc* test, as indicated in the Figure legends. In all of the reported statistical analysis effects were designated as significant if the *p* value was <0.05.

## Results

### *In silico* Prediction of Binding of Bisphenols to Nuclear Receptors

Prediction of the binding of BPA, BPAF, and BPS to the nuclear receptors AR and GR relevant for RACK1 expression was performed with two *in silico* programs: Endocrine Disruptome (Kolšek et al., 2014) and VirtualToxLab (Vedani et al., 2009; Vedani et al., 2012). These data are shown in Tables 1, 2. Both *in silico* tools returned interactions of the three bisphenols with AR. Endocrine Disruptome suggested a moderately high probability to the agonist conformation of AR for BPA and BPS, whereas a low binding probability for BPAF is indicated. Moreover, a high binding probability to the antagonist conformation of AR for BPA and BPAF, and a moderately high probability for BPS is suggested (Table 1). VirtualToxLab indicated binding concentrations to AR at 139 and 143 nM for BPA and BPAF, respectively, and at the higher concentration of 2.3 µM for BPS (Table 2). The same analysis on Endocrine Disruptome was also performed for GR, that returned a moderately low probability of binding to the agonist conformations for all of bisphenols, and low for the antagonist conformation (Table 1). VirtualToxLab indicated that BPA and BPAF can interact with GR at 341 and 363 nM respectively and BPS at 10.5 µM (Table 2).

**TABLE 1:** Endocrine Disruptome calculated free energies and predicted probabilities for bisphenols binding to nuclear receptors.

Nuclear	Conform	Bisphenols binding					
Receptor		BPA		BPAF		BPS	
		Free energy (kcal/mol)	Prediction	Free energy (kcal/mol)	Prediction	Free energy (kcal/mol)	Prediction
Androgen	Agonist	−8.4	Moderately high	−7.1	Low	−8.2	Moderately high
	Antagonist	−8.5	High	−9.3	High	−8.1	Moderately high
Glucocorticoid	Agonist	−7.6	Moderately low	−8.3	Moderately low	−7.6	Moderately low
	Antagonist	−7.5	Low	−7.9	Low	−7.1	Low

**TABLE 2:** VirtualToxLab predicted toxic potentials and concentrations (maximum 100 μM) for bisphenols binding to nuclear receptors.

	Bisphenols binding concentration		
	BPA	BPAF	BPS
**Nuclear Receptor**			
Androgen	139 nM	143 nM	2.3 µM
Glucocorticoid	341 nM	363 nM	10.5 µM
**Toxic potential**			
**Range (0–1)**	0.490	0.509	0.352

Overall, based on the binding affinities indicated by these analyses, a role of BPA, BPAF and BPS in affecting RACK1 expression could be suggested, since they show an interaction profile with the nuclear receptors demonstrated to regulate RACK1 transcription, namely AR and GR (Racchi et al., 2017). However, since these softwares return a possible binding affinity for both the agonist and antagonist nuclear receptor structures for all bisphenols, this suggest that cellular context need to be considered to elucidate their actual mechanism of action. Noteworthy, a range of concentrations for bisphenols treatments were then selected based on this analysis in order to investigate potential non-monotonic dose-response effect commonly observed for hormones and hormone-active substances.

### Bisphenols A Inhibits Receptor for Activated C Kinase 1 Expression and Blocks Immune Activation

**FIGURE 1:**
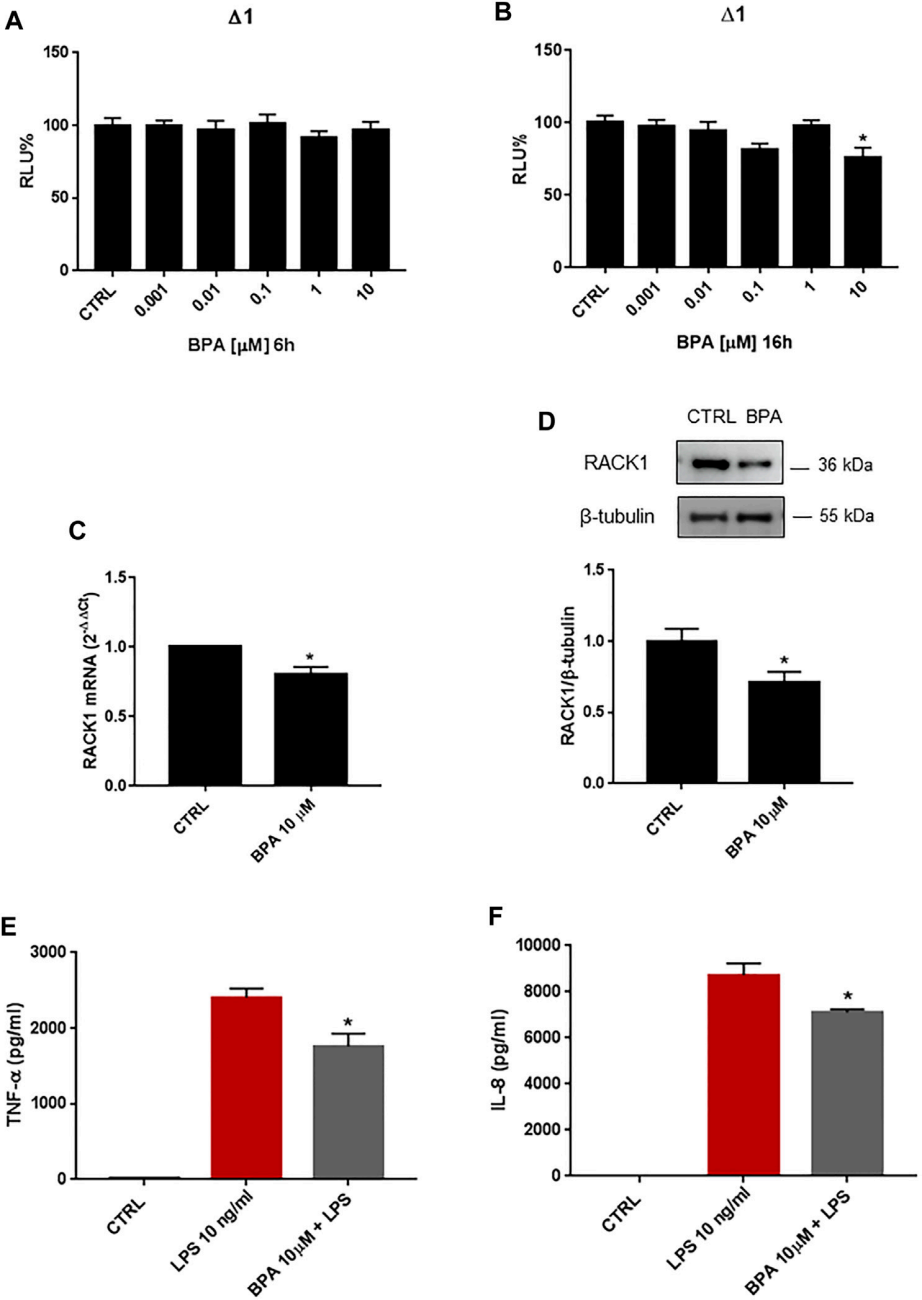
Effects of BPA on *RACK1* expression and immune activation. (**A–B)** THP-1 cells transiently transfected with the Δ1 construct were treated for 6 h **(A)** or 16 h (**B**) with increasing concentrations of BPA (0.001–10 μM) or DMSO as vehicle control (CTRL). Cells were lysed and luciferase activity was measured as described in “Materials and Methods” section. Luciferase activities are expressed as RLU% respected to non-treated construct (considered as 100%). Results are expressed as mean ± SEM, *n* = 3 independent experiments performed in quadruplicate. Statistical analysis was performed with Dunnett’s multiple comparison test with *p < 0.05 vs. control (CTRL). **(C-D)** THP-1 cells treated for 18 h **(C)** or 24 h (**D**) with 10 μM BPA or DMSO as vehicle control (CTRL). **(C)** mRNA levels evaluated by qPCR (endogenous reference, 18S). (**D**) The image is a representative Western blot. RACK1 protein levels evaluated by Western blotting, normalized to β-tubulin expression. Each value represents the mean ± SEM *n* = 3 independent experiments. Significance was set at *p* < 0.05 by the Student’s *t*-test (*p < 0.05). **(E–F)** THP-1 cells treated with 10 ng/ml LPS without and with 10 μM BPA or DMSO as vehicle control (CTRL). Secretion of cytokines TNF-α **(E)** and IL-8 **(F)** evaluated by sandwich ELISAs. Statistical analysis was performed with Student’s *t*-test with *p < 0.05 vs LPS alone.

BPA is a diphenylmethane derivative that is used in the production of polycarbonate plastics and epoxy resins (Monneret, 2017) and is known to have hormone-like, xenoestrogen and estrogen-mimicking properties (Murata and Kang, 2018). Given its wide industrial use and these known endocrine-disrupting features, BPA exposure in humans has raised concerns, particularly for its possible influence on the immune system (Buoso et al., 2020c). Based on our previous data on the effects of EDCs on RACK1 expression and altered immune responses, we investigated the effects of BPA on RACK1 expression by reporter luciferase activity using the human *RACK1* gene promoter, mRNA by qPCR, and protein levels by Western blotting. THP-1 cells were treated for 6, 16, 18 or 24 h with increasing concentrations of BPA (0.001–10 μM) or DMSO as vehicle control, according to literature data (Couleau et al., 2015). These timings were selected based on previous experiments as being optimal to investigate dehydroepiandrosterone (DHEA) and cortisol-induced *RACK1* transcriptional activity, mRNA and protein expression (Buoso et al., 2011) as well as the effects of EDCs on RACK1 (Buoso et al., 2017a; Buoso et al., 2020b). While 6 h of BPA treatment did not affect *RACK1* gene promoter activity at any of the concentrations tested here (Figure 1A), 10 μM BPA for 16 h induced significant *RACK1* down-regulation (Figure 1B). Accordingly, 10 μM BPA also induced significant decreases in RACK1 mRNA (Figure 1C) and protein (Figure 1D) levels at 18 and 24 h, respectively. Hormone-related *RACK1* expression is known to correlate with TNF-α and IL-8 cytokine secretion (Racchi et al., 2017). Upon 10 µM BPA treatment, we observed this correlation for both TNF-α (Figure 1E) and IL-8 (Figure 1F). It is important to note that BPA alone did not induce cytokine production (data not shown).

### Unmasking the Bisphenol A Effects on Receptor for Activated C Kinase 1 Expression

**FIGURE 2:**
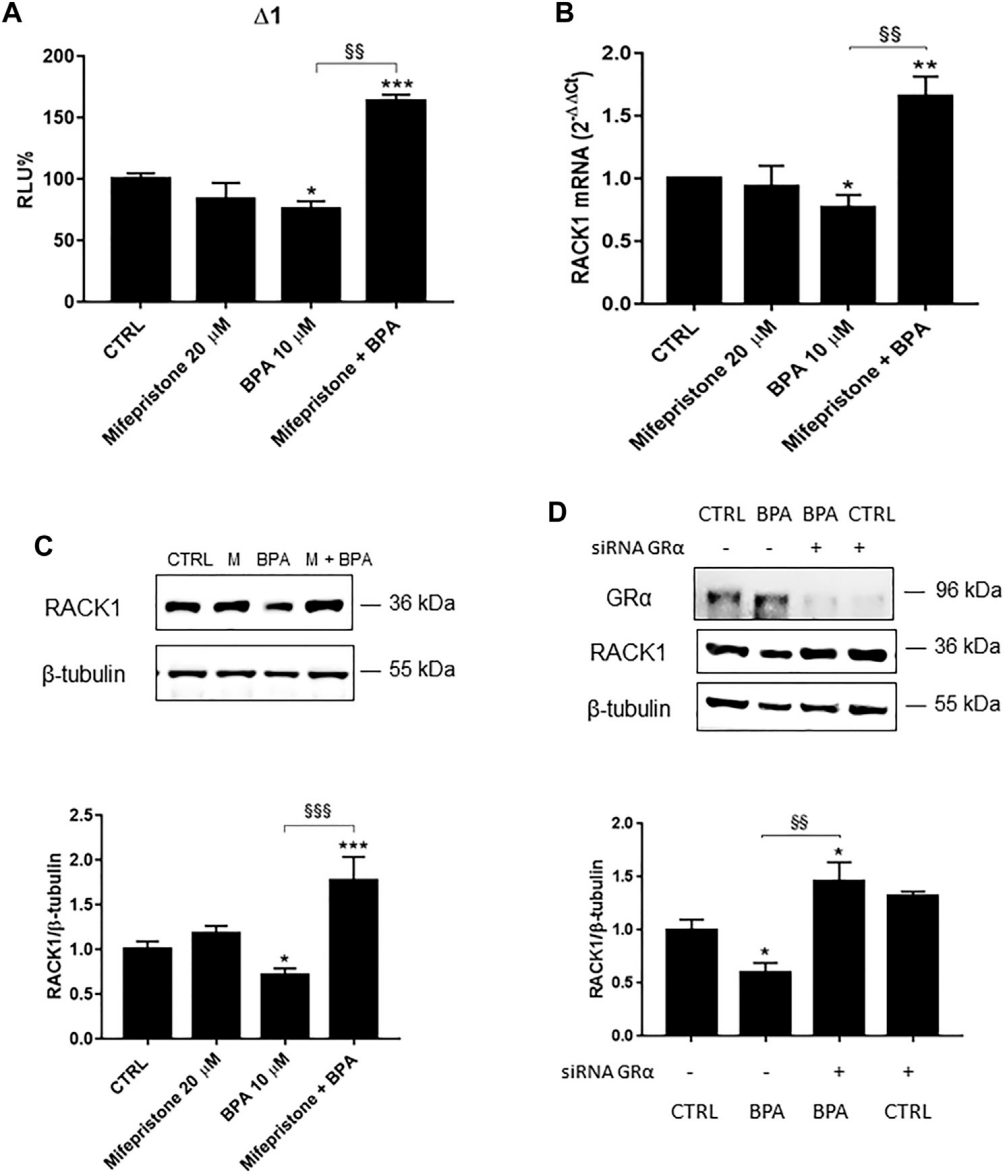
Role of GRα in BPA-induced *RACK1* regulation. **(A)** THP-1 cells transiently transfected with the Δ1 construct were pre-treated for 30 min with 20 μM mifepristone, then with 10 μM BPA for 16 h or DMSO as vehicle control (CTRL). Cells were lysed and luciferase activity was measured as described in “Materials and Methods” section. Luciferase activities are expressed as RLU% respected to non-treated construct (considered as 100%). Results are expressed as mean ± SEM, *n* = 3 independent experiments performed in triplicate. Statistical analysis was performed with Tukey’s multiple comparison test with *p < 0.05, ***p < 0.001 vs. control (CTRL) and ^§§^ *p* < 0.01 vs. BPA 10 μM. **B-C** THP-1 cells pre-treated for 30 min with 20 μM mifepristone were treated for 18 h (**B**) or 24 h **(C)** with 10 μM BPA or DMSO as vehicle control (CTRL). **(B)** mRNA levels evaluated by qPCR (endogenous reference, 18S). **(C)** The image is a representative Western blot. RACK1 protein levels evaluated by Western blotting, normalized to β-tubulin expression. Each value represents the mean ± SEM, *n* = 3 independent experiments. Statistical analysis was performed with Tukey’s multiple comparison test with *p < 0.05, **p < 0.01, ***p < 0.001 vs control (CTRL) and ^§§^ *p* < 0.01, ^§§§^ *p* < 0.001 vs BPA 10 μM. **(D)** THP-1 cells silenced for 48 h with GRα-directed siRNA were treated for 24 h with 10 μM BPA or DMSO as vehicle control (CTRL). The image is a representative Western blot. RACK1 and GRα protein levels evaluated by Western blotting, normalized to β-tubulin expression. Statistical analysis was performed with Tukey’s multiple comparison test with *p < 0.05 vs control (CTRL) and ^§§^ *p* < 0.01 vs. BPA-treated nonsilenced cells.

We previously reported that BPA induced a low but significant RACK1 down-regulation (Figure 1) and this could be due to its ability to bind AR in an antagonist conformation (Table 1). In line with this consideration, our previous data demonstrated that RACK1 basal expression is positively regulated by androgens and AR silencing induced a significant down-regulation of RACK1 expression (Racchi et al., 2017). However, since BPA and other bisphenols are known to bind and activate different nuclear receptors, including GRα (Prasanth et al., 2010; Zhang et al., 2017; Buoso et al., 2020c) and that GR binding to RACK1 promoter induces its down-regulation (Racchi et al., 2017), we cannot exclude GR involvement in the observed BPA-mediated effect on RACK1 expression. Therefore, to unmask AR-mediated BPA effects, we pre-treated THP-1 cells with mifepristone, a well-known GR inhibitor (Bertagna et al., 1984), in order to exclude GR involvement and highlight BPA antagonistic activity for AR. As shown in Figures 2A–C, in the cells treated with mifepristone and 10 μM BPA, there was significant up-regulation of *RACK1* promoter activity (Figure 2A) and increased mRNA (Figure 2B) and protein (Figure 2C) levels compared to 10 μM BPA alone. The same unmasking effect on BPA was also observed with lower tested concentrations (Supplementary Figure S1A). In addition, these data were mirrored by 10 μM BPA treatment in GRα-silenced THP-1 cells (Figure 2D). Therefore, these data show that BPA effects on RACK1 expression are more likely correlated with its agonist profile for GRα rather than an antagonist profile for AR. This is in accordance with BPA moderately low affinity for GR agonist conformation (Table 1) that correlates with the slight BPA effect on RACK1.

### Effects of Bisphenol AF on Receptor for Activated C Kinase 1 Expression

**FIGURE 3:**
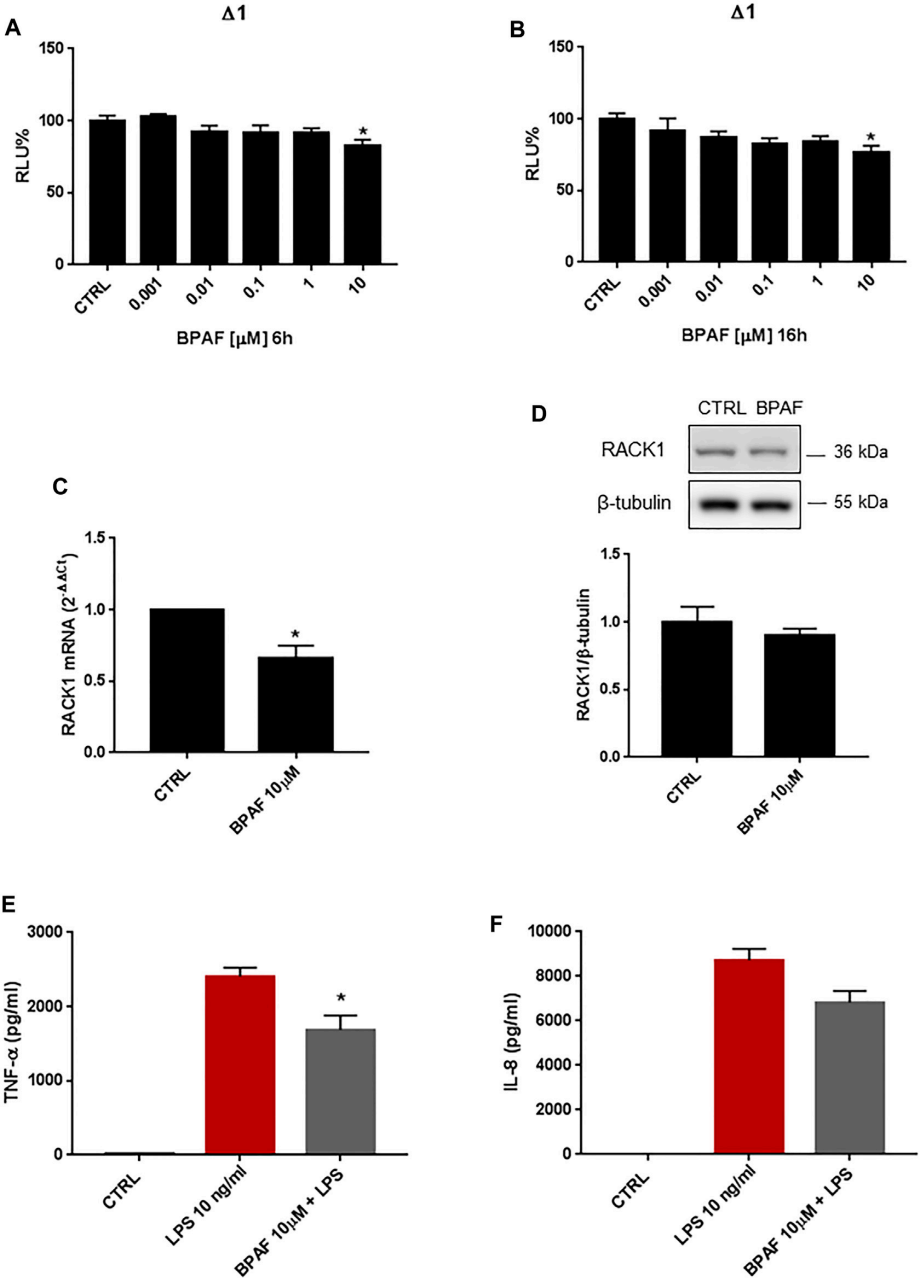
Effects of BPAF on *RACK1* expression and immune activation. **(A, B)** THP-1 cells transiently transfected with the Δ1 construct were treated for 6 h **(A)** or 16 h **(B)** with increasing concentrations of BPAF (0.001–10 μM) or DMSO as vehicle control (CTRL). Cells were lysed and luciferase activity was measured as described in “Materials and Methods” section. Luciferase activities are expressed as RLU% respected to non-treated construct (considered as 100%). Results are expressed as mean ± SEM, *n* = 3 independent experiments performed in quadruplicate. Statistical analysis was performed with Dunnett’s multiple comparison test with *p < 0.05 vs control (CTRL). **(C–D)** THP-1 cells treated for 18 h **(C)** or 24 h **(D)** with 10 μM BPAF or DMSO as vehicle control (CTRL). **(C)** mRNA levels evaluated by qPCR (endogenous reference, 18S). **(D)** The image is a representative Western blot. RACK1 protein levels evaluated by Western blotting, normalized to β-tubulin expression. Each value represents the mean ± SEM *n* = 3 independent experiments. Significance was set at *p* < 0.05 by the Student’s *t*-test (*p < 0.05). **(E–F)** THP-1 cells treated with 10 ng/ml LPS without and with 10 μM BPAF or DMSO as vehicle control (CTRL). Secretion of cytokines TNF-α **(E)** and IL-8 **(F)** evaluated by sandwich ELISAs. Statistical analysis was performed with Student’s *t*-test with *p < 0.05 vs LPS alone.

BPAF is a bisphenol analog that has replaced BPA in several industrial settings. Increasing evidence has suggested, however, that BPAF is characterized by greater estrogenic properties compared to BPA. THP-1 cells were treated for 6, 16, 18 and 24 h with increasing concentrations of BPAF (0.001–10 μM) or DMSO as vehicle control. As shown in Figures 3A,B, 10 μM BPAF treatment induced a statistically significant decrease of *RACK1* transcriptional activity at 6 and 16 h, respectively. These effects on *RACK1* gene promoter activation were reflected by RACK1 mRNA (Figure 3C) but were not appreciable at protein level (Figure 3D). The slight reduction of RACK1 promoter activity appears to be sufficient to affect mRNA but not RACK1 protein and, although a slight decrease was observed, it did not reach statistical significance. This is most likely related to the turnover time of the protein in THP-1 cells (Corsini et al., 2002; Del Vecchio et al., 2009).

Since a strong correlation between RACK1 expression and TNF-α release has been demonstrated, in accordance with the slight but significant RACK1 down-regulation observed after BPAF treatment, a low decrease in LPS-induced TNF-α secretion is reported (Figure 3E). Accordingly, BPAF did not affect IL-8 secretion compared to LPS treatment alone (Figure 3F). It is important to note that BPAF alone did not induce cytokine production (data not shown).

### Unmasking Bisphenol AF Effects on Receptor for Activated C Kinase 1 Expression

**FIGURE 4:**
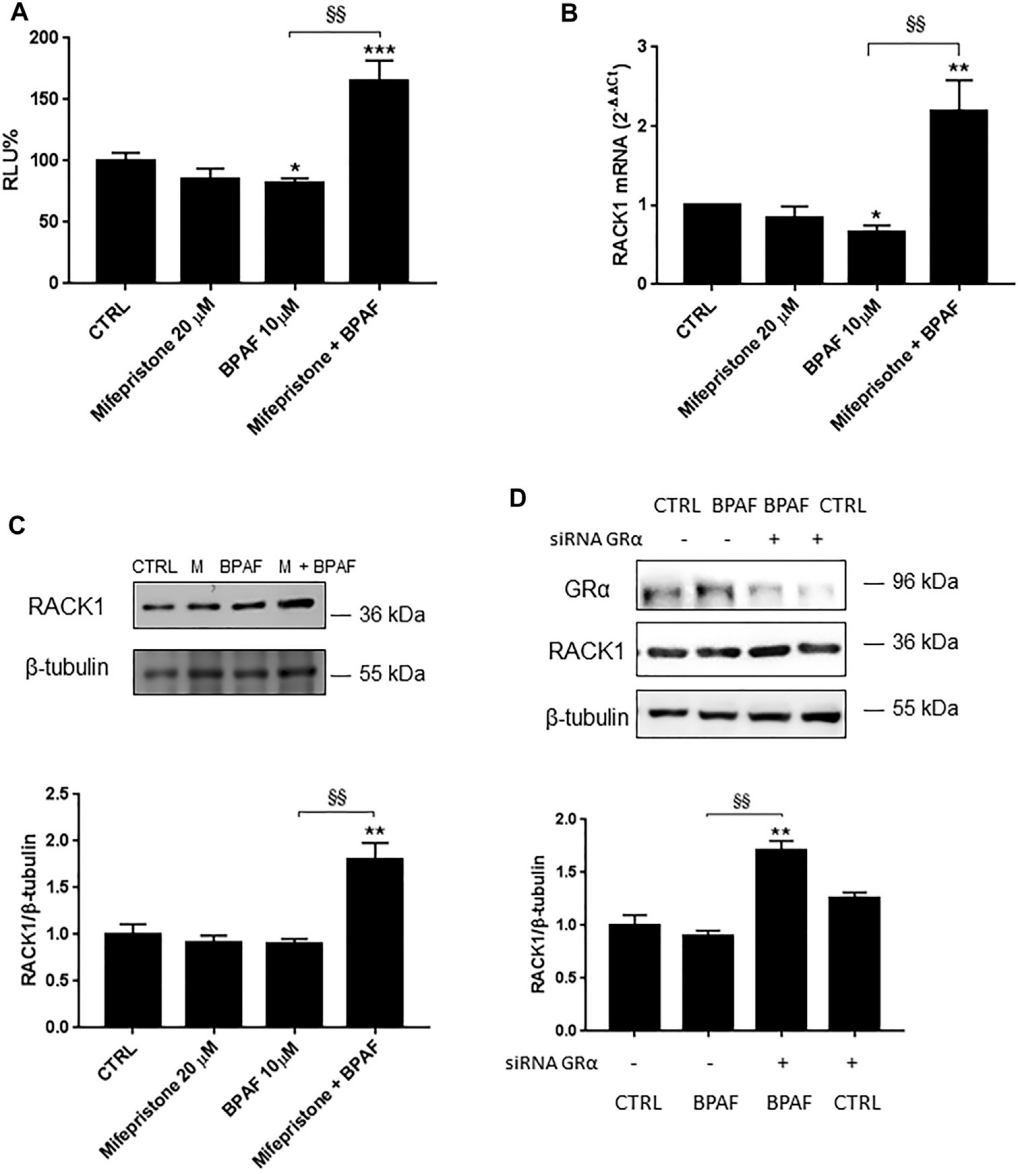
Role of GRα in BPAF-induced *RACK1* regulation. **(A)** THP-1 cells transiently transfected with the Δ1 construct were pre-treated for 30 min with 20 μM mifepristone, then with 10 μM BPAF for 16 h or DMSO as vehicle control (CTRL). Cells were lysed and luciferase activity was measured as described in “Materials and Methods” section. Luciferase activities are expressed as RLU% respected to non-treated construct (considered as 100%). Results are expressed as mean ± SEM, *n* = 3 independent experiments performed in triplicate. Statistical analysis was performed with Tukey’s multiple comparison test with *p < 0.05, ***p < 0.001 vs. control (CTRL) and ^§§^ *p* < 0.01 vs. BPAF 10 μM. **(B–C)** THP-1 cells pre-treated for 30 min with 20 μM mifepristone were treated for 18 h **(B)** or 24 h **(C)** with 10 μM BPAF or DMSO as vehicle control (CTRL). **(B)** mRNA levels evaluated by qPCR (endogenous reference, 18S). **(C)** The image is a representative Western blot. RACK1 protein levels evaluated by Western blotting, normalized to β-tubulin expression. Each value represents the mean ± SEM, *n* = 3 independent experiments. Statistical analysis was performed with Tukey’s multiple comparison test with *p < 0.05, **p < 0.01 vs control (CTRL) and ^§§^ *p* < 0.01 vs BPAF 10 μM. **(D)** THP-1 cells silenced for 48 h with GRα-directed siRNA were treated for 24 h with 10 μM BPAF or DMSO as vehicle control (CTRL). The image is a representative Western blot. RACK1 and GRα protein levels evaluated by Western blotting, normalized to β-tubulin expression. Statistical analysis was performed with Tukey’s multiple comparison test with **p < 0.01 vs control (CTRL) and ^§§^ *p* < 0.01 vs. BPAF-treated nonsilenced cells.

Like BPA, BPAF can bind AR in an antagonist conformation (Table 1) and/or activate several other nuclear hormone receptors, including GRα (Kojima et al., 2019; Skledar et al., 2019). Therefore, to unmask the possible AR-mediated BPAF effects, we pre-treated THP-1 cells with mifepristone and, as for BPA, there was significant up-regulation of *RACK1* promoter activity (Figure 3A) and increased mRNA (Figure 3B) and protein (Figure 3C) levels compared to 10 μM BPAF alone. The same unmasking effect on BPAF was also observed with lower tested concentrations (Supplementary Figure S1B). These data were mirrored by 10 μM BPAF treatment in the GRα-silenced THP-1 cells (Figure 4D) thus indicating that BPAF shows a mild agonist profile for GRα rather than an antagonist profile for AR, in line with its moderately low affinity for GR agonist conformation (Table 1).

Altogether, these data show that GRα inhibition or silencing can unmask another BPAF-related effect, which suggests that similar to BPA, BPAF can induce alterations in RACK1 levels via GRα signaling as well as through other cellular pathways.

### Role of Androgen Receptor and NF-κB in the Unmasked Bisphenol A and Bisphenol AF-Induced Receptor for Activated C Kinase 1 Expression

**FIGURE 5:**
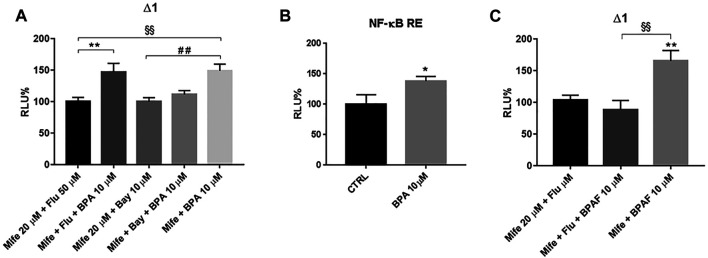
Unmasked BPA and BPAF effects on *RACK1* promoter. THP-1 cells were transiently transfected with the Δ1 construct **(A, C)** or the pGL4.32 [luc2P/NF-κB-RE/Hygro] vector reporter construct **(B)**. **(A, C)** After transfection, cells were pre-treated for 30 min with 20 μM mifepristone (Mife) with or without 50 μM flutamide (Flut) **(A, C)** and 1 h with 10 μM Bay 11–7085 (Bay) **(A)**, then treated with 10 μM BPA **(A)** or 10 μM BPAF **(C)** for 16 h. Cells were lysed, and luciferase activity was measured as described in “Materials and Methods” section. Luciferase activities are expressed as RLU% respected to non-treated construct (considered as 100%). Results are expressed as mean ± SEM, *n* = 3 independent experiments performed in triplicate. Statistical analysis was performed with Tukey’s multiple comparison test with **p < 0.01 vs Mife 20 μM + Flu 50 μM, ^§§^ *p* < 0.01 vs Mife 20 μM + Flu 50 μM and ^##^ *p* < 0.01 vs Mife 20 μM + Bay 10 μM. **B** After transfection, cells were treated for 16 h with 10 μM BPA or DMSO as vehicle control (CTRL). Results are expressed as mean ± SEM, *n* = 3 independent experiments performed in triplicate. Significance was set at *p* < 0.05 by the Student’s *t*-test (*p < 0.05).

Our data show that hampering BPA binding to GRα through either receptor inhibition or gene silencing, another BPA-related effect could be unmasked and its antagonist profile for AR can be excluded. In addition, BPA agonist activity on AR can also be ruled out since in immune context and in THP-1 cells, we demonstrated that not only RACK1 has a basal regulation controlled by androgens and AR that counteracts GR activity, but also that treatment with androgenic compounds results in a significant up-regulation of RACK1 (Buoso et al., 2017a; Buoso et al., 2017b; Buoso et al., 2020b; Buoso et al., 2020c; Racchi et al., 2017; Buoso et al., 2017c). Therefore, due to its known estrogenic-like features, as reported in the literature, BPA can bind and activate GPER (previously known as GPR30) (Périan and Vanacker, 2020). GPER activation has been reported to induce RACK1 increase by AR ligand independent activation (Buoso et al., 2020b). Therefore, with flutamide treatment, we investigated the possible mechanism that links the mifepristone-unmasked BPA effects on *RACK1* up-regulation with hormone response element (HRE) identified in the *RACK1* promoter region (Racchi et al., 2017). Cells were treated for 1 h with 50 μM flutamide, 30 min with 20 μM mifepristone and then for 16 h with 10 μM BPA or the DMSO vehicle control for luciferase activity. As shown in Figure 5A, BPA could still significantly induce RACK1 promoter activation in the presence of flutamide, suggesting that BPA masked positive effect on RACK1 is not AR-dependent. The same mechanism was confirmed with BPA lower tested concentrations (Supplementary Figure S1A). Since GPER is known to activate PI3K/Akt signaling cascade (Buoso et al., 2020b) and consequently NF-κB, we assessed BPA-mediated NF-κB activation due to the presence of c-Rel binding sites in RACK1 promoter region (Del Vecchio et al., 2009). To this purpose, we treated THP-1 cells with the IκB degradation inhibitor Bay 11–7085, along with mifepristone. As shown in Figure 5A, RACK1 promoter activity was completely and significantly prevented by Bay 11–7085 treatment, suggesting that BPA masked positive effect on RACK1 is related to the presence of c-Rel site according to our previous data (Buoso et al., 2013; Racchi et al., 2017; Masi et al., 2020). To confirm whether BPA can activate NF-κB, we transfected THP-1 cells with a NF-κB specific luciferase reporter vector (Fagiani et al., 2020). As shown in Figure 5B, BPA treatment resulted in a significant activation of the NF-κB-recognized vector, confirming that BPA unmasked effect on RACK1 could be NF-κB-mediated.

The same considerations were made for BPAF but, as shown in Figure 5C, flutamide completely blocked mifepristone-unmasked BPAF effect on RACK1 transcriptional activity, demonstrating the role of GPER-activated AR in its observed effects. The same mechanism was confirmed with BPAF lower tested concentrations (Supplementary Figure S1B).

### Bisphenol A and Bisphenol AF G-Protein-Coupled Estrogen Receptor Molecular Docking

**FIGURE 6:**
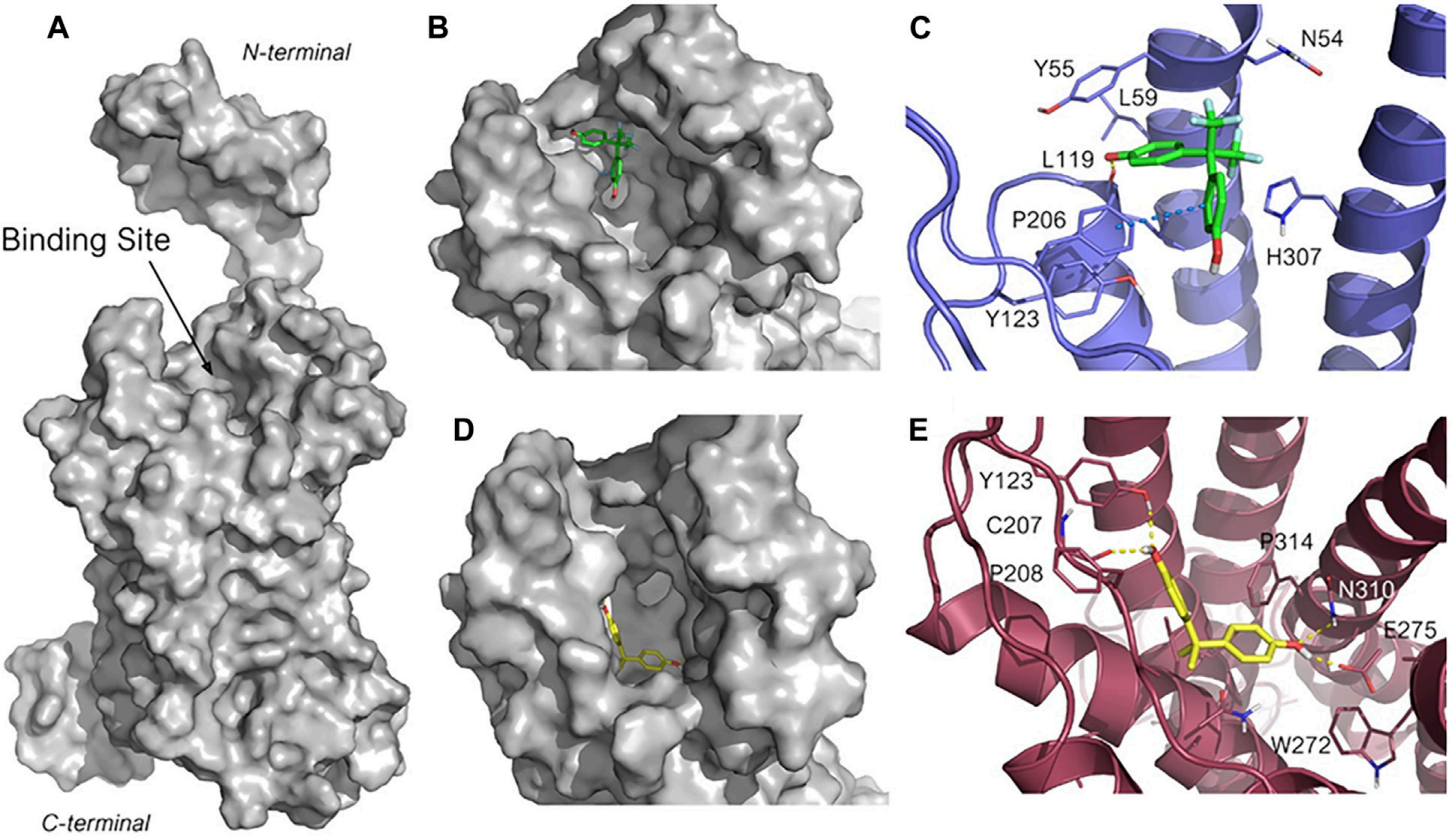
Predicted binding mode for BPAF and BPA in GPER ligand binding site. **(A)** Structure of GPER obtained through homology modeling by using the web server GPCR-I-TASSER (HG ID: HG0714). The putative binding site is indicated. **(B, C)** Close-up of the binding site highlighting the different sub-pockets occupied by BPAF (**B**, in green sticks carbons) and BPA (**D**, in yellow sticks carbon). **(D, E)** Highest ranked pose for BPAF (**D**, in green stick carbons) and BPA (**E**, in orange sticks carbons) within the GPER binding site. The 3D structure of GPER is represented in blue cartoon **(D)** and warmpink cartoon **(E)**, and the key amino acid residues interacting with the ligand are represented in lines. The heteroatoms are color-coded: oxygen in red, nitrogen in blue, sulfur in yellow and fluorine in pale green. The H-bonding and the π–π stacking are represented in yellow, and blue dotted lines, respectively.

To investigate BPA and BPAF different mechanism of action, molecular docking calculations were then performed to gain structural insight into their interactions with GPER. As the crystallographic structure of GPER is not available at present, its most recent structure that was generated by homology modeling (HG ID: HG0714) was downloaded from the web server GPCR-ITASSER (Zhang and Zhang, 2010; Zhang et al., 2015). GPCR-ITASSER is a computational suite derived from the parent I-TASSER package, and it is the most accurate homology modeling software customized for G-protein-coupled receptors (GPCRs). These two bisphenols were then docked into the putative ligand binding pocket of GPER, as suggested by a previous computational study (Rosano et al., 2016) (Figure 6A). Ten docking poses for each ligand were generated. The structures of the most favored docked GPER/ligand complexes are shown in Figures 6B–E. Interestingly, although BPA and BPAF have high structural similarity, they bind into two different clefts of the wide GPER binding site (Figures 6B,C). BPAF interacts with the most superficial portion of the receptor site and its binding pose is highly conserved among the first seven poses with the lowest docking score (in the range of −6.08 to −5.92). A highly negative docking score is associated with a more suitable receptor-ligand binding, and the narrow range of docking score is indicative of the robustness of the predicted binding mode. In the lowest energetic complex, one aromatic ring is allocated into a hydrophobic pocket delimited by Leu59, Tyr55 and Leu119, whereas the phenolic hydroxyl is involved in a hydrogen bond with the carbonyl backbone of Leu119. The second aromatic ring is instead involved in a face-to-edge π-π bond with Phe206. Lastly, the two polar trifluoromethyl (-CF3) groups are located within a polar cleft of the receptor binding site, partially exposed to the solvent, and delimited by Gln54, and His307 (Figure 6D).

Conversely, as depicted in Figure 6C, BPA is predicted to bind into a deeper and less exposed sub-pocket of GPER binding site and the predicted poses are well reproduced in all the GPER-BPA complexes. BPA is accommodated within a portion of the receptor binding site delimited by non-polar amino acids such as Met133, Leu137, Met141, Phe208, Ala209, and Phe314. This hydrophobic pocket is more favorable to host lipophilic molecules such BPA than BPAF, thus accounting for the different orientation observed for the two assessed ligands. In addition, both phenolic hydroxyls of BPA participate into a network of hydrogen bonds involving Tyr123, the backbone carbonyl of Cys207, Glu275, and Gln310, and that contribute to tight the binding of the BPA with the receptor (Figure 6E). These data support the experimental evidence reported above and suggest that, upon binding, BPA and BPAF trigger different GPER conformational changes that lead to the activation of different signaling pathways.

### Bisphenol S Induces Receptor for Activated C Kinase 1 Expression

**FIGURE 7:**
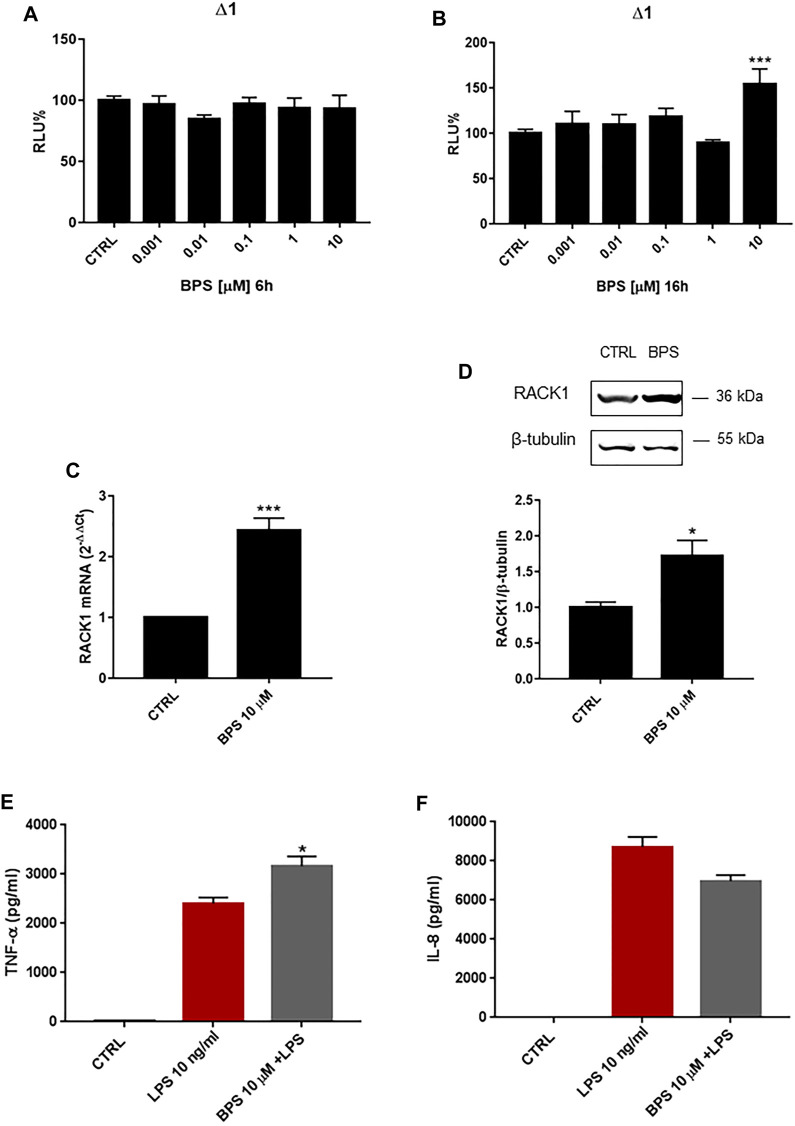
Effects of BPS on *RACK1* expression and immune activation. **(A–B)** THP-1 cells transiently transfected with the Δ1 construct were treated for 6 h **(A)** or 16 h **(B)** with increasing concentrations of BPS (0.001–10 μM) or DMSO as vehicle control (CTRL). Cells were lysed and luciferase activity was measured as described in “Materials and Methods” section. Luciferase activities are expressed as RLU% respected to non-treated construct (considered as 100%). Results are expressed as mean ± SEM, *n* = 3 independent experiments performed in quadruplicate. Statistical analysis was performed with Dunnett’s multiple comparison test with ***p < 0.001 vs. control (CTRL). **(C–D)** THP-1 cells treated for 18 h **(C)** or 24 h **(D)** with 10 μM BPS or DMSO as vehicle control (CTRL). **(C)** mRNA levels evaluated by qPCR (endogenous reference, 18S). **(D)** The image is a representative Western blot. RACK1 protein levels evaluated by Western blotting, normalized to β-tubulin expression. Each value represents the mean ± SEM *n* = 3 independent experiments. Significance was set at *p* < 0.05 by the Student’s *t*-test (*p < 0.05, ***p < 0.01). **(E–F)** THP-1 cells treated with 10 ng/ml LPS without and with 10 μM BPS or DMSO as vehicle control (CTRL). Secretion of cytokines TNF-α **(E)** and IL-8 **(F)** evaluated by sandwich ELISAs. Statistical analysis was performed with Student’s *t*-test with *p < 0.05 vs LPS alone.

Finally, we investigated the effects of another BPA analogue, BPS, on *RACK1* expression. As for the other bisphenols, THP-1 cells were treated for 6, 16, 18 and 24 h with increasing concentrations of BPS (0.001–10 μM) or DMSO as vehicle control. As shown in Figures 7A 6 h BPS treatment did not alter *RACK1* promoter activity at any of the concentrations tested, while 16 h treatment induced significant *RACK1* up-regulation only at 10 μM BPS (Figure 7B). In agreement with this, there was a statistically significant increase in RACK1 mRNA expression after 18 h of treatment with 10 μM BPS (Figure 7C), which was mirrored by increased RACK1 protein levels after 24 h of treatment (Figure 7D). Similar to BPA and BPAF, we also investigated TNF-α and IL-8 release for BPS-treated and LPS-stimulated THP-1 cells (Figures 7E,F). Consistent with the increase in *RACK1* expression, there was a significant increase in TNF-α secretion for 10 μM BPS, although no increase in the release of IL-8 was reported. It is important to note that BPS alone did not induce cytokine production (data not shown).

### Role of Androgen Receptor in Bisphenol S-Induced Receptor for Activated C Kinase 1 Expression

**FIGURE 8:**
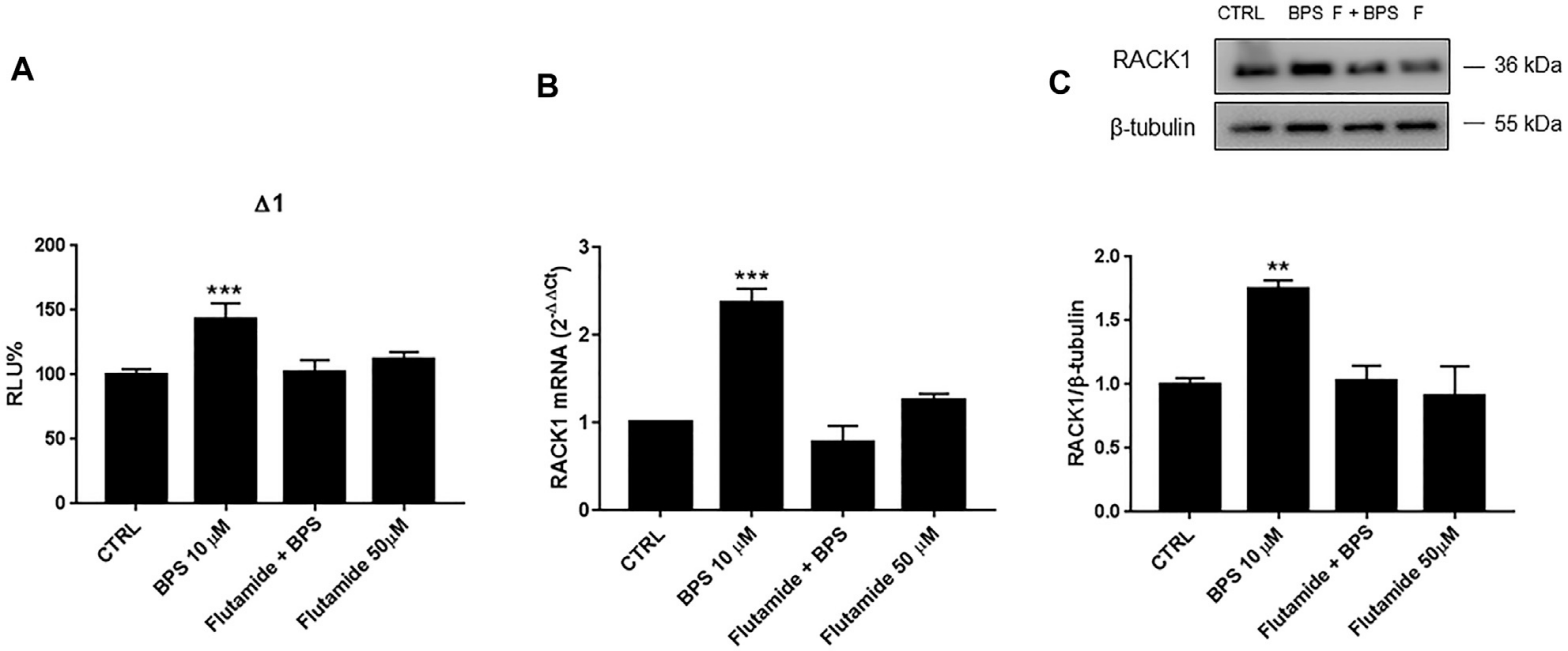
Role of AR in BPS-induced *RACK1* regulation. **(A)** THP-1 cells transiently transfected with the Δ1 construct were pre-treated for 1 h with 50 μM flutamide, then with 10 μM BPS for 16 h or DMSO as vehicle control (CTRL). Cells were lysed and luciferase activity was measured as described in “Materials and Methods” section. Luciferase activities are expressed as RLU% respected to non-treated construct (considered as 100%). Results are expressed as mean ± SEM, *n* = 3 independent experiments in triplicate. Statistical analysis was performed with Dunnett’s multiple comparison test with ***p < 0.001 vs control (CTRL). **(B, C)** THP-1 cells pre-treated for 1 h with 50 μM flutamide for 18 h **(B)** or 24 h **(C)** with 10 μM BPS or DMSO as vehicle control (CTRL). **(B)** mRNA levels evaluated by qPCR (endogenous reference, 18S). **(C)** The image is a representative Western blot. RACK1 protein levels evaluated by Western blotting, normalized to β-tubulin expression. Each value represents the mean ± SEM, *n* = 3 independent experiments. Statistical analysis was performed with Dunnett’s multiple comparison test with **p < 0.01; ***p < 0.001 vs control (CTRL).

Due to the BPS up-regulation of *RACK1* expression, we investigated whether this increase was AR-mediated. THP-1 cells were, therefore, pre-treated with flutamide for 1 h. As shown in Figures 8 10 μM BPS alone induced a significant increase in *RACK1* promoter activity (Figure 8A), and the consequent up-regulation of RACK1 at both the mRNA (Figure 8B) and protein (Figure 8C) levels. Then, in line with the previous data here, flutamide pre-treatment completely abolished BPS-mediated *RACK1* up-regulation at the promoter (Figure 8A), and also at the mRNA (Figure 8B) and protein (Figure 8C) levels. To investigate a possible masked effect on BPS we performed the same experimental setting as for BPA and BPAF. However, mifepristone treatment did not unmask any other BPS effect on RACK1 expression. (Supplementary Figure S2). Hence, these data confirmed that these effects of BPS on *RACK1* are mediated by AR and are in line with BPS moderately high AR agonist profile reported in Table 1.

## Discussion

**FIGURE 9:**
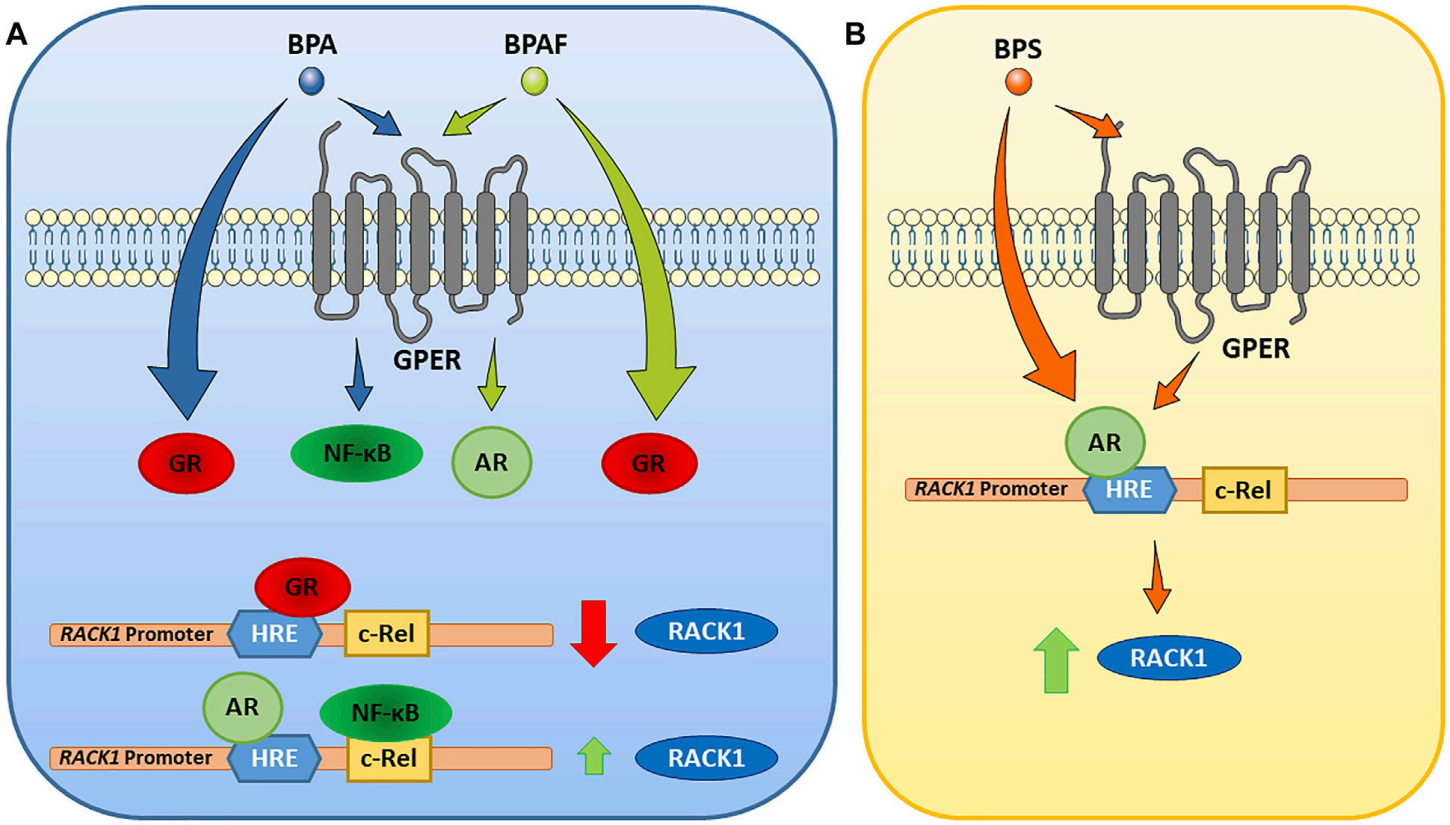
Proposed mechanisms of action of BPA, BPAF and BPS on *RACK1* expression. Following the data, our model indicates that BPA and BPAF act through glucocorticoid receptor (GR), and G-protein-coupled estrogen receptor (GPER). BPS acts predominantly through androgen receptor (AR), although GPER involvement cannot be excluded (see text for details). AR, androgen receptor; BPA, bisphenol A; BPAF; bisphenol AF; BPS, bisphenol S; GPER, G-protein-coupled estrogen receptor; GR, glucocorticoid receptor; HRE, hormone response element; NF-κB, nuclear factor κB; RACK1, receptor for activated C kinase 1.

In our previous published manuscripts, we have demonstrated that, in immune context, RACK1 expression can be regulated by glucocorticoids, androgens and estrogen-active compounds (Buoso et al., 2017a; Racchi et al., 2017; Buoso et al., 2020b; Corsini et al., 2021). All began from the observation of a GRE sequence on the human RACK1 promoter, which is responsible of cortisol and corticosteroids action at the transcriptional level resulting in the down-regulation of RACK1 expression (Buoso et al., 2011; Corsini et al., 2014a; Corsini et al., 2014b). The anti-glucocorticoid effect of dehydroepiandrosterone (DHEA), through the conversion to active androgens, has an opposite effect on RACK1 expression and on the regulation of PKC activity involved in immune cell activation. A more specific indication derived from data demonstrating that DHEA effect on RACK1 expression could be completely prevented using flutamide, as an AR antagonist or AR silencing, thus resulting in no response to DHEA in terms of RACK1 expression and LPS-induced cytokine production. Therefore, modulation of AR is a key step in the mechanism supporting RACK1 expression (Racchi et al., 2017; Corsini et al., 2021). Indeed, it is noteworthy that AR and GR can interact at the transcriptional level since they recognize a similar palindromic sequence usually termed as a canonical androgen/glucocorticoid response element (ARE/GRE) (Pihlajamaa et al., 2015). Since RACK1 plays a pivotal role in immune cell activation (Corsini et al., 1999; Corsini et al., 2005; Corsini et al., 2014a; Corsini et al., 2014b; Buoso et al., 2017b; Buoso et al., 2017c; Racchi et al., 2017), and a complex hormonal balance is involved in the control of its expression (Buoso et al., 2011; Buoso et al., 2017b; Buoso et al., 2017c; Racchi et al., 2017; Corsini et al., 2021), it could represent a useful tool for the screening of EDCs and their immunotoxicity (Buoso et al., 2017a; Racchi et al., 2017; Buoso et al., 2020b; Corsini et al., 2021). Indeed, it is emerging evidence for a role of EDCs in the immune system dysfunction (Bansal et al., 2018; Corsini et al., 2018; Nowak et al., 2019). Hence, the aim of the present study was to investigate whether the bisphenols A, AF and S regulate RACK1 expression and LPS-induced TNF-α and IL-8 production. These immune parameters were selected since we previously demonstrated to be dependent upon RACK1/PKCβ activation (Corsini et al., 1999; Corsini et al., 2005; Buoso et al., 2011; Corsini et al., 2014a; Corsini et al., 2014b; Buoso et al., 2017b; Buoso et al., 2017c; Racchi et al., 2017). Our present data showed that RACK1 was down-regulated upon exposure to BPA and BPAF and this modulation was mirrored by a low but statistically significant decrease of the aforementioned cytokines release. Because of the observed reduction of RACK1 expression, mifepristone treatment was used to discriminate whether BPA and BPAF showed AR antagonism or GR agonism profile as emerged by our *in silico* analysis through the Endocrine Disruptome. In this regard, mifepristone allowed not only to reveal that BPA and BPAF act as GR agonists but also to unmask further correlated effects, which were detected at lower concentrations, reflecting levels of exposed subjects (European Food Safety Authority, 2017; Ribeiro et al., 2019). The unmasking effect of mifepristone was also confirmed by GRα silencing. Due to unmasked RACK1 up-regulation and BPA and BPAF known estrogen-like properties, to better dissect their molecular mechanism, the involvement of GPER in bisphenol-mediated RACK1 modulation was investigated according to our previous results (Buoso et al., 2020b). Based on literature data on GPER-activated signaling cascade, flutamide treatment was used to assess GPER-mediated AR ligand independent activation upon BPA and BPAF exposure (Buoso et al., 2020b). In this experimental setting, while BPAF up-regulated RACK1 expression through the same mechanism that we previously demonstrated for the estrogen-active compound diethylstilbestrol (DES), BPA induced RACK1 expression through a different mechanism that involves NF-κB (Buoso et al., 2013; Racchi et al., 2017; Masi et al., 2020). The rational being that four c-Rel binding sites were identified in the human RACK1 promoter (Del Vecchio et al., 2009; Buoso et al., 2013). c-Rel is a member of the NF-κB transcription factor family, whose involvement in RACK1 regulation was demonstrated in different cellular context (Buoso et al., 2013; Masi et al., 2020), including cells of immune origin using LPS (Del Vecchio et al., 2009). Our molecular docking confirmed that BPA and BPAF different mechanisms of action could be explained due to their diverse binding mode to GPER, suggesting a biased agonism for this membrane receptor (Figure 9A).

Regarding BPS, our data showed that RACK1 expression was up-regulated and this modulation was mirrored by a low but statistically significant increase of TNF-α. According to RACK1 hormonal regulation literature, we investigated AR involvement in its increased expression. Flutamide treatment confirmed that BPS effects are mediated by its AR agonism in line with our *in silico* predictions with Endocrine Disruptome. However, since BPS is a BPA analog and displays a weak estrogenic activity (Buoso et al., 2020c), we cannot totally exclude a partial GPER involvement in AR activation although we can confirm that its effects are mediated by AR (Figure 9B).

Altogether, these data demonstrated that RACK1 is an useful tool to unravel the interfering effects of EDCs on endocrine-regulated cellular processes, especially thanks to RACK1 expression modulation and its downstream effects on the innate immune response. In particular, this work reveals that the regulation of RACK1 expression can be used to investigate the complex molecular mechanism of EDCs that involve different receptors, both nuclear and membrane bound, including bisphenols. In addition, specific inhibition and gene silencing of well-known RACK1 transcriptional modulators can be also used to unmask other EDCs molecular effects. Therefore, this work perfectly fits in our research frame, because we exploited this same molecular unmasking approach as we previously did for estrogenic-active compounds DES and zearalenone (ZEA) (Buoso et al., 2020b). Indeed, although RACK1 promoter region lacks an estrogen responsive element, its ability to be regulated through a non-genomic cascade activated by GPER allows RACK1 to be used to screen well-known estrogenic-like molecules, such as these bisphenols.

In term of hazard identification, our results indicate that high concentrations of exposure to BPS predispose cells to an enhanced immune response, which, depending on the context, could be detrimental, i.e. it can favor the onset of autoimmune diseases, allergic reactions to unrelated antigens, misregulated inflammation to mention some possible consequences. Conversely, BPA and BPAF are associated in a decreased immunostimulation, with a greater effect of BPA compared to BPAF, that should be considered indicative of immunotoxicity. In this regard, different BPA occupational exposure studies performed on Chinese workers at BPA manufacturers and epoxy resin reported in urine samples ≤10 μM BPA concentrations, suggesting that in these workers immunological implications can occur due to BPA exposure (He et al., 2009; Li et al., 2010; Miao et al., 2014; Liu et al., 2015; Miao et al., 2015). However, usual exposure concentrations of BPA and BPAF do not modify RACK1 expression and do not trigger immune response. This is due to their ability to interact and activate different receptors, thus creating a balance, which results in no detectable effects, in line with CLARITY-BPA study observations (Li et al., 2018) and other literature data (Kimber, 2017). In this context, it is noteworthy that our data unravel how differences in the amount of bisphenols-interacting receptors or impaired endocrine responses in the exposed subjects could be at the basis of the unmasked bisphenols effects, which may be responsible to the discrepancy of published data on bisphenols, especially with BPA. Hence, immune features of subjects exposed to typical bisphenols concentration could be essential to predict acute and/or chronic effects on the immune system that ultimately do not depend only on the compounds themselves.

To conclude, we propose an *in vitro* strategy where as a screening, molecular modelling and docking simulation to assess the affinity for steroid hormones receptors together with the RACK1 promoter activity should be the initial step, followed by RACK1 mRNA and/or protein expression to confirm that changes in the promoter activity have an impact on cellular RACK1 level, and finally, the physiological consequences of its modulation, can be investigated by evaluating immune functions. The other advantage in the use of RACK1 is that its expression can capture the complex interplay of transcriptional and non-transcriptional events associated with exposure to hormonally active compounds targeting more than one hormonal system and the resulting biological consequence, as we previously demonstrated (Buoso et al., 2011; Buoso et al., 2017b; Buoso et al., 2017c; Buoso et al., 2020b). Results warrant further analysis of panels of EDCs differently targeting steroid receptors.

## Data Availability

The raw data supporting the conclusions of this article will be made available by the authors, without undue reservation, to any qualified researcher.
