# Supplementary figures and images for: Effects of Bisphenols on RACK1 Expression and Their Immunological Implications in THP-1 Cells
Source: Front Pharmacol. 2021 Sep 21;12:743991. doi: 10.3389/fphar.2021.743991 (PMC8490885; doi:10.3389/fphar.2021.743991)

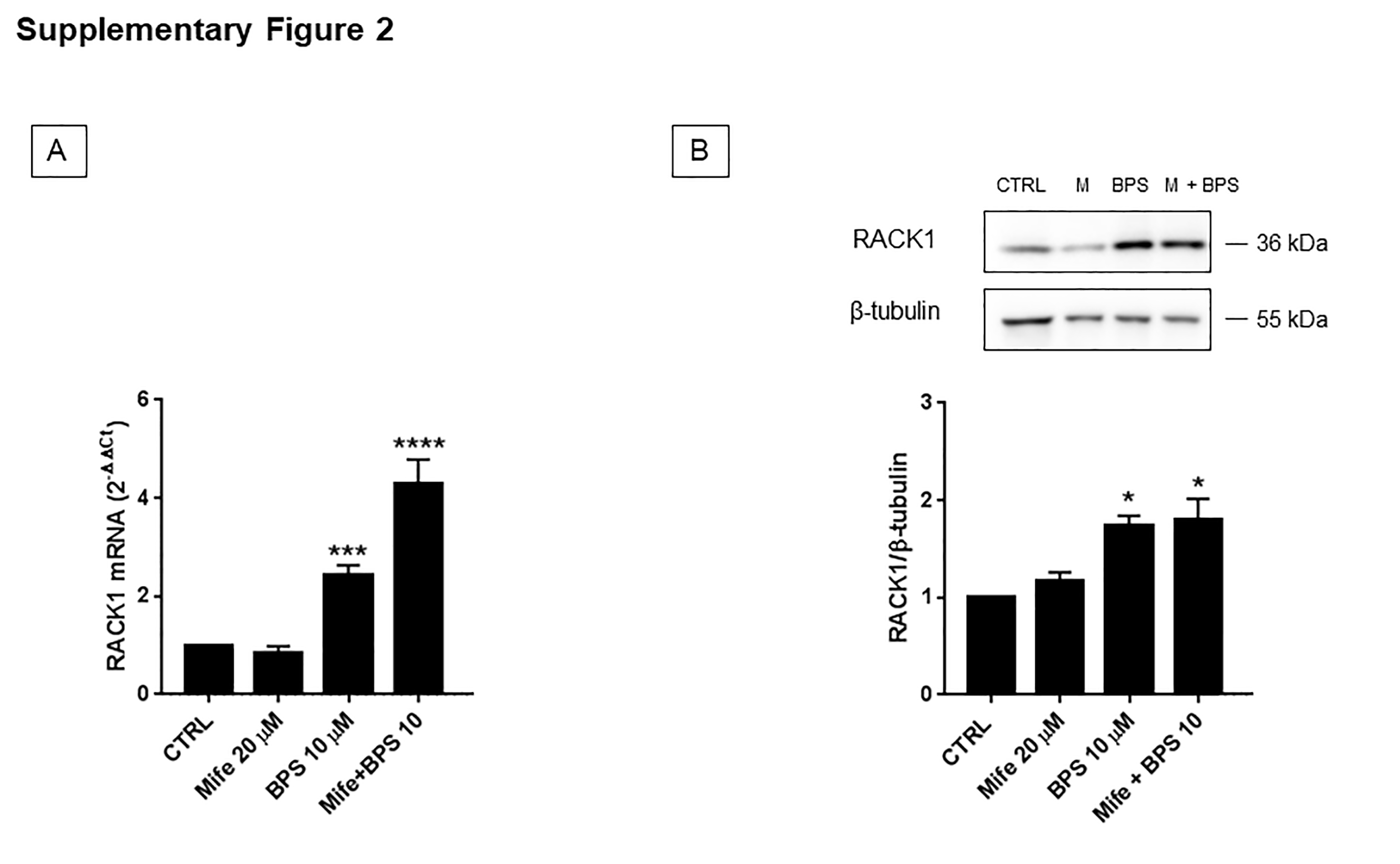

Supplement: Supplementary file 1 [file Image2.TIF]

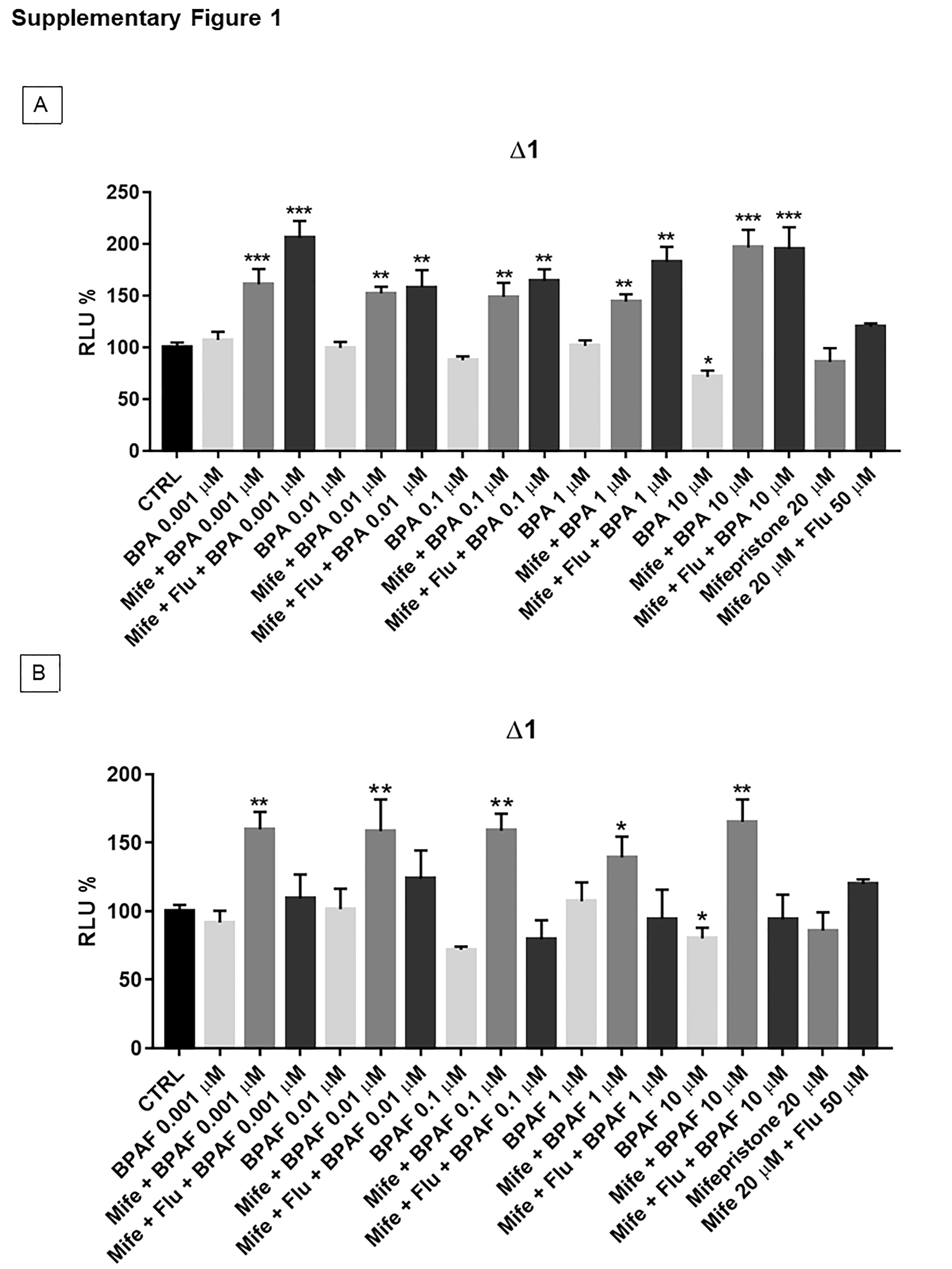

Supplement: Supplementary file 2 [file Image1.TIF]
